# Proton Therapy With Concurrent Chemotherapy for Thoracic Esophageal Cancer: Toxicity, Disease Control, and Survival Outcomes

**DOI:** 10.14338/IJPT-22-00021.1

**Published:** 2022-12-19

**Authors:** Michael S. Rutenberg, Bradford S. Hoppe, Jason S. Starr, Ziad Awad, Mathew Thomas, Christopher G. Morris, Perry Johnson, Randal H. Henderson, Jeremy C. Jones, Bharatsinh Gharia, Steven Bowers, Herbert C. Wolfsen, Sunil Krishnan, Stephen J. Ko, Hani M. Babiker, Romaine C. Nichols

**Affiliations:** 1 Department of Radiation Oncology, Mayo Clinic, Jacksonville, FL, USA; 2 Division of Hematology/Oncology, Mayo Clinic, Jacksonville, FL, USA; 3 Department of Surgery, University of Florida College of Medicine, Jacksonville, FL, USA; 4 Department of Cardiothoracic Surgery, Mayo Clinic, Jacksonville, FL, USA; 5 Department of Radiation Oncology, University of Florida College of Medicine, Jacksonville, FL, USA; 6 Department of Medicine, University of Florida College of Medicine, Jacksonville, FL, USA; 7 Department of Gastroenterology and Hepatology, Mayo Clinic Jacksonville, FL, USA

**Keywords:** radiation therapy, particle therapy, cancer outcomes, cancer side effects

## Abstract

**Purpose:**

When treating esophageal cancer with radiation therapy, it is critical to limit the dose to surrounding structures, such as the lung and/or heart, as much as possible. Proton radiation therapy allows a reduced radiation dose to both the heart and lungs, potentially reducing the risk of cardiopulmonary toxicity. Here, we report disease control, survival, and toxicity outcomes among patients with esophageal cancer treated with proton radiation therapy and concurrent chemotherapy (chemoradiation therapy; CRT) with or without surgery.

**Materials and Methods:**

We enrolled 17 patients with thoracic esophageal carcinoma on a prospective registry between 2010 and 2021. Patients received proton therapy to a median dose of 50.4-GyRBE (range, 50.4–64.8) in 1.8-Gy fractions.Acute and late toxicities were graded per the Common Terminology Criteria for Adverse Events, version 4.0 (US National Cancer Institute, Bethesda, Maryland). In addition, disease control, patterns of failure, and survival outcomes were collected.

**Results:**

Nine patients received preoperative CRT, and 8 received definitive CRT. Overall, 88% of patients had adenocarcinoma, and 12% had squamous cell carcinoma. With a median follow-up of 2.1 years (range, 0.5–9.4), the 3-year local progression-free, disease-free, and overall survival rates were 85%, 66%, and 55%, respectively. Two patients (1 with adenocarcinoma and 1 with squamous cell carcinoma) recurred at the primary site after refusing surgery after a complete clinical response to CRT. The most common acute nonhematologic and hematologic toxicities, respectively, were grades 1 to 3 esophagitis and grades 1 to 4 leukopenia, both affecting 82% of patients. No acute cardiopulmonary toxicities were observed in the absence of surgical resection. Reagarding surgical complications, 3 postoperative cardiopulmonary complications occurred as follows: 1 grade 1 pleural effusion, 1 grade 3 pleural effusion, and 1 grade 2 anastomotic leak. Two severe late CRT toxicities occurred: 1 grade 5 tracheoesophageal fistula and 1 grade 3 esophageal stenosis requiring a feeding tube.

**Conclusion:**

Proton radiation therapy is a safe, effective treatment for esophageal cancer with increasing evidence supporting its role in reducing cardiopulmonary toxicity.

## Introduction

Attaining the optimal curative outcomes among patients with locally advanced esophageal cancer typically requires a multimodality treatment approach, including chemotherapy, radiation therapy, and surgery. Yet, managing this malignancy can present multidisciplinary treatment challenges because the esophagus is crucial to maintaining nutrition [1, 2]. Furthermore, the location of the esophagus—nestled between the lungs and immediately posterior to the heart—renders surgery and radiation therapy challenging due to the risks of treatment injury to these critical organs [3–5]. Fortunately, over the past several decades, advances in surgical and radiation therapy techniques and a better understanding of the optimal management of esophageal cancer have improved outcomes, including disease control and cure rates [6–9]. As such, the importance of minimizing treatment-related toxicities and maintaining quality of life has become increasingly relevant. Proton therapy represents a technological advancement that can facilitate optimal disease control while reducing risks to nearby critical organs.

The dosimetric benefit of proton therapy compared with photon-based radiation therapy (including 3-dimensional conformal radiation therapy, intensity-modulated radiation therapy [IMRT], and volumetric modulated arc therapy) has been repeatedly demonstrated through its significant reduction of radiation dose to the heart and lungs [10–13]. In contrast to IMRT or volumetric modulated arc therapy, proton therapy does not require a compromise in heart dose to reduce the lung dose or vice versa; sparing of both is readily achievable with protons. A recent single-institution, randomized phase II trial comparing proton therapy to IMRT showed a reduction in acute and postoperative cardiopulmonary complications [14]. Despite encouraging dosimetric and early clinical data, relatively few clinical reports describe outcomes among patients treated with proton therapy for esophageal cancer. Most available clinical outcomes come from a single institution (MD Anderson Cancer Center) [14–17]. Here, we describe a collaborative effort between the University of Florida and Mayo Clinic Florida to analyze outcomes in patients treated with proton therapy with concurrent chemotherapy (chemoradiation therapy; CRT) for esophageal cancer. We report acute and late treatment-related complications, disease control, and survival outcomes.

## Materials and Methods

### Patient Population

We reviewed the medical records of 17 consecutive patients treated with proton therapy and concurrent chemotherapy between August 2010 and June 2021 for biopsy-proven thoracic esophageal adenocarcinoma or squamous cell carcinoma (SCC). Patients provided informed consent for enrollment on an institutional review board–approved outcomes tracking protocol. All patients received proton therapy (at the University of Florida) with concurrent chemotherapy and with or without surgical resection at either the University of Florida or Mayo Clinic Florida. Patients with cervical esophageal cancer, prior history of thoracic or abdominal radiation therapy, or metastatic disease were excluded, as were patients who received radiation therapy without concurrent chemotherapy. No patient received postoperative radiation therapy.

### Workup and Treatment

All patients completed staging with upper endoscopy and endoscopic ultrasound with computed tomography (CT) of the chest, abdomen, and pelvis and/or positron emission tomography–CT. The staging was performed according to the 8th edition of the *American Joint Committee on Cancer* (AJCC) staging manual [18]. For patients treated before the AJCC 8th edition, restaging was done for purposes of this research to report our data using a uniform staging system across all patients.

Radiation planning included a 4-dimensional CT scan to assess the respiratory motion of the target. All patients were treated supine, and most had their arms elevated on a wing board with a vacuum-based immobilization device. However, 2 patients received treatment with their arms down due to the discomfort with their arms elevated. Target contours were delineated on the average-phase CT registered to the staging CT or positron emission tomography–CT. The internal gross tumor volume (IGTV) was generated on the average phase CT with modifications to account for motion seen on each of the individual 4-dimensional CT phases. The internal target volume (ITV) was generated by adding a 3- to 4-cm anatomically constrained proximal and distal mucosal expansion and a 1-cm radial expansion around the esophagus to the internal gross tumor volume. The gross lymph nodes were contoured and expanded by 5 to 10 mm to generate a gross nodal ITV. The mediastinal lymph nodes and celiac lymph nodes were electively covered at the discretion of the treating radiation oncologist. Elective celiac lymph node coverage was generally included for patients with distal esophagus or gastroesophageal junction primaries. Elective mediastinal lymph node coverage was considered for patients with upper- and mid-thoracic primaries and grossly positive periesophageal lymph nodes at or near the level of the mediastinal lymph node stations. If grossly involved mediastinal lymph nodes were present, mediastinal lymph node stations adjacent to the involved lymph node were covered electively. Lymph nodes were considered grossly involved if they were enlarged (≥ 10 mm), had adverse-appearing features (rounded with loss of normal fatty hilum), or showed F-fluorodeoxyglucose uptake above background levels on positron emission tomography imaging [19]. Boost targets included the internal gross tumor volume of the primary and nodal disease with a 5- to 10-mm expansion. Each ITV was expanded 5 mm isotropically to the planning target volumes. Target coverage goals included 99% and 95% of each ITV and planning target volumes, respectively, covered by the prescription dose. The **Figure** shows representative treatment plans with isodose distributions. **Table 1** shows heart and lung planning goals and median values achieved among all patients.

**Figure.**
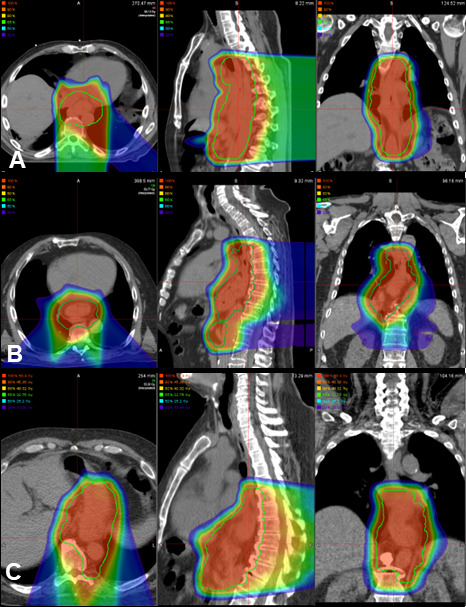
Proton beam arrangements used among the cohort. (A) Two fields with double-scattered posterior-anterior and left posterior oblique fields. (B) Three fields with double-scattered posterior anterior, left posterior oblique, and right posterior oblique fields. (C) Two fields with pencil-beam scattering using left and right posterior oblique fields with volumetric repainting.

**Table 1.** Heart and lung dose constraints and dose-volume histogram parameters for proton radiation therapy (N = 17).

**Structure**	**Planning goal**	**Median (range)**
Lung		
Mean lung dose	≤ 20 Gy	6.5 GyRBE (1.8–9.7)
Lung V5, %	≤ 50	29.3 (8.0–42.2)
Lung V10, %	≤ 40	21.2 (6.3–34.9)
Lung V20, %	≤ 25	10.9 (3.3–24.2)
Heart		
Mean heart dose	≤ 26 Gy	7.8 GyRBE (1.0–18.0)
Heart V40, %	≤ 50	7.4 (0–23.0)
Heart V5, %	≤ 65	24.1 (4.4–74.0)

**Abbreviations:** Lung V5, volume of normal lung receiving 5 Gy; Lung V10, volume of normal lung receiving 10 Gy; Lung V20, volume of normal lung receiving 20 Gy; Heart V40, volume of normal heart receiving 40 Gy; Heart V5, volume of normal heart receiving 5 Gy.

Patients typically underwent sequential treatment planning with an initial dose of 45 GyRBE to the gross disease and elective lymph nodes and a boost ranging from 5.4 to 19.8 GyRBE (total dose range, 50.4 Gy to 64.8 GyRBE) depending on the plan for surgical resection versus definitive CRT. Three patients received treatment to 50.4 GyRBE in a single-treatment volume. All treatment was delivered using 1.8-GyRBE fractions. All but 1 patient received treatment with double-scattered protons. Custom brass apertures and acrylic tissue compensators were generated for each treatment field for use with passive-scattered treatment plans. Treatment planning usually included either 2 (n = 8) or 3 (n = 8) fields for each target volume; 1 patient was treated with 4 fields. The most common field arrangements included the following: (1) left and right posterior oblique pairs (LPO and RPO), (2) a posterior-anterior field and 2 posterior oblique fields (LPO and RPO), or (3) a posterior-anterior field with a single lateral oblique field (either RPO or LPO) (**Figure**). One patient treated with pencil-beam scattering (PBS) received intensity-modulated proton therapy delivered to planning target volumes 1 using a 3-field approach (posterior-anterior, LPO, RPO) with volumetric repainting for each field followed by a 2-field boost (RPO, LPO) with volumetric repainting. For this case, robust optimization was used with 4% range uncertainty and 4-mm setup uncertainty.

All patients received concurrent chemotherapy during proton radiation. Sixteen patients received weekly carboplatin and paclitaxel, while 1 received cisplatin and fluorouracil.

### Disease Control and Toxicity Outcomes

Local recurrence was defined as disease recurrence in the initial target volume (within the primary site or treated regional lymph nodes). Regional recurrence was defined as a recurrence in the regional lymph nodes outside the treatment target volume. Distant failures were defined as nonregional lymph node, osseous, or visceral metastases. The date to local, regional, or distant recurrence was calculated from the date of diagnosis. Overall survival was calculated from the date of diagnosis to the date of death or last follow-up. Disease-free survival was calculated from the date of diagnosis to the date of recurrence, death, or last follow-up. Local recurrence-free survival was calculated from the date of diagnosis to the date of local recurrence, death, or last follow-up.

Acute and late treatment–related toxicities were prospectively graded per the National Cancer Institute's Common Terminology Criteria for Adverse Events, version 4.0. Acute hematologic toxicities were recorded from complete blood count analyses obtained at baseline before beginning CRT (within 1 week of beginning CRT), during CRT (weekly or biweekly), and up to 4 weeks after CRT. Complete blood count analyses without differential cell counts were obtained for many patients during CRT, limiting our ability to assess additional hematology-related toxicities (eg, lymphopenia, neutropenia). Only hemoglobin, hematocrit, leukocyte count, and platelet counts were available for all patients to assess hematologic toxicity.

### Statistical Analysis

SAS version 9.4 and JMP Pro version 16.1.0 were used for statistical analysis (SAS Institute, Cary, NC). Basic descriptive statistics are provided for this series. Medians rather than means were used to estimate the center of a continuous distribution to avoid the effect of outliers. The Kaplan-Meier product limit estimator was used to estimate overall survival. The cumulative incidence method with intercurrent death treated as a competing risk was used to assess local control and disease-free survival.

## Results

### Patent and Tumor Characteristics

Seventeen patients (15 men, 2 women) with thoracic esophageal carcinoma who received proton therapy at our institution enrolled in this study, while 2 patients declined to participate. The median follow-up for all patients was 2.1 years (range, 0.5–9.4 years). For surviving patients, the median follow-up was 2.7 years (range, 0.7–9.4 years). The median age at diagnosis was 67.4 years (range, 44.2–79.9 years). Fifteen patients had adenocarcinoma, and 2 had squamous cell carcinoma. Patient, disease, and treatment characteristics are detailed in **Table 2,** while patient comorbidities are outlined in the **Supplemental Table**. Eight patients (47%) received definitive CRT without surgical resection as follows: 3 refused surgery, and 5 were inoperable (either medically or due to disease extent). Among the 5 inoperable patients, 1 patient had T4b disease with an invasion of the left mainstem bronchus. Before receiving definitive CRT, this patient received 8 months of induction chemotherapy with a good local response and resolution of the bronchial invasion (without metastatic progression). Nine patients (53%) received preoperative CRT as part of trimodality therapy.

**Table 2.** Patient, disease, and treatment characteristics (N = 17).

**Characteristic**	**Value**
Sex, n (%)	
Male	15 (88)
Female	2 (12)
Age at diagnosis, median (range), y	67.4 (44.2–79.9)
Tumor location, n	
Upper thoracic	1
Mid thoracic	6
Distal thoracic or gastroesophageal junction	10
Tumor histology ,n	
Adenocarcinoma	15
Squamous cell carcinoma	2
Tumor (T) and nodal (N) stage, n	
T2N0	2
T2N1	3
T3N0	5
T3N1	3
T3N2 or N3	3
T4N0	1
Treatment	
Definitive CRT, n	8
Preoperative CRT, n	9
Radiation dose, median (range) GyRBE	50.4 (50.4–64.8)
Chemotherapy regimen (patients), n (%)	
Carboplatin and paclitaxel	13 (76)
Cisplatin and fluorouracil	2 (12)
Cisplatin and docetaxel	1 (6)
Xeloda and oxaliplatin	1 (6)

Abbreviations: CRT; chemoradiation therapy.

### Disease Control and Survival

The 3-year cumulative incidences of local control, progression-free survival, and overall survival were 85%, 66%, and 55%, respectively (**Supplemental Figure**). In total, 5 patients experienced disease recurrence: 2 with local recurrence and 3 with distant metastases. Both patients with local failures refused surgery following CRT after an initial complete clinical treatment response. One patient had cT3N2M0 SCC and received 54 GyRBE with concurrent carboplatin and paclitaxel, which recurred 7 months after CRT. The patient refused salvage surgery and was alive with progressive disease at the last follow-up. The other patient with a local recurrence was treated for T3N0 adenocarcinoma and received 64.8 GyRBE with concurrent carboplatin and paclitaxel. The patient was initially planned for preoperative CRT but decided against surgery during treatment; therefore, the dose was increased from a planned 50.4 GyRBE to 64.8 GyRBE. The patient developed a local recurrence 23 months after CRT and underwent salvage esophagectomy. The patient is alive with no evidence of disease 5 months after salvage surgery. Three patients developed distant metastatic disease between 1 and 14 months after completing CRT: 2 with osseous metastases and 1 with pulmonary metastases.

### Treatment-Related Toxicities

#### Acute toxicities

No acute nonhematologic grade 4 to 5 CRT-associated toxicities were observed. The most common treatment-related complication was esophagitis. Of patients, 58% (n = 10) had grade 1 to 2 esophagitis and 24% (4 patients) developed grade 3 esophagitis (1 requiring hospitalization and 3 requiring enteral nutrition). Two patients (12%) developed grade 3 dehydration, and 6% developed grade 3 nausea and vomiting (1 patient each). **Table 3** details acute nonhematologic treatment-related toxicities.

**Table 3.** Acute nonhematologic^a^ and hematologic^b^ toxicity (N = 17).^b^

**Toxicity**	**Patients, n**			
	**Grade 1**	**Grade 2**	**Grade 3**	**Grade 4**
Nonhematologic				
Esophagitis	5	5	4	0
Fatigue	3	2	0	0
Dermatitis	4	1	0	0
Insomnia	2	0	0	0
Nausea	2	3	1	0
Vomiting	2	2	1	0
Weight loss	3	1	0	0
Gastric ulcer	0	1	0	0
Dehydration	0	1	2	0
Dyspnea	1	0	0	0
Hematologic				
Anemia	6	6	1	0
Leukopenia	5	4	3	2
Thrombocytopenia	4	0	0	0

Note: No acute grade 5 toxicities were observed.

a ≤ 90 days of completing chemoradiation therapy.

b ≤ 30 days of completing chemoradiation therapy.

Our cohort's most common hematologic toxicity was leukopenia, affecting 83% of patients (n = 14). Nine patients (53%) had grade 1 to 2 leukopenia; 3 patients (18%) had grade 3 leukopenia; and 2 patients (12%) developed grade 4 leukopenia. No grade 5 hematologic toxicities were observed (**Table 3**).

Among the 9 patients who underwent surgical resection following CRT, 8 experienced acute surgical complications. Two patients had 1 grade 1 toxicity each (pleural effusion and cough), and 2 more had 1 grade 2 toxicity each (anastomotic leak and deep vein thrombosis). Three patients (33%) experienced a total of 4 grade 3 postoperative complications, including 1 patient with pleural effusions requiring chest tubes, 1 patient with vocal cord paralysis, 1 patient with pyloric stenosis (requiring dilation), and 1 patient experienced a 22% decrease in weight. No grade 4-5 postoperative complications occurred.

#### Late toxicities

Two grade 3 or higher late toxicities were attributable to CRT (12%), including 1 grade 5 event due to a tracheoesophageal fistula. This grade 5 event occurred 7 months after CRT in the setting of an esophageal stent placed 4 months after definitive CRT to a total dose of 64.8 GyRBE. No evidence of disease was found at the time of death. The other severe late CRT-related toxicity included 1 grade 3 esophageal stenosis requiring enteral nutrition. **Table 4** details late CRT-associated toxicities.

**Table 4.** Late chemoradiation therapy–associated toxicity.^a^

**Toxicity**	**Grade**	**Patients, n**
Pyloric stenosis	3	1
Tracheoesophageal fistula	5	1^b^
Esophageal stenosis	2	1
	3	1
Atrial fibrillation	2	1
Esophageal hemorrhage	1	1^c^

a > 90 days from chemoradiation therapy start.

b Likely due to esophageal stent.

c While anticoagulated after percutaneous coronary intervention (for left anterior descending artery atherosclerosis—outside the radiation therapy field) nearly 10 years after chemoradiation therapy; resolved without intervention.

## Discussion

Progress in the management of localized esophageal cancer has been slow and incremental. However, in the past decade, several seminal studies have clarified the optimal treatment of esophageal cancer and helped refine our treatment approaches. The CROSS and NEOCRTEC5010 trials confirmed the overall survival benefit of preoperative CRT compared with surgery alone for both SCC and adenocarcinoma of the esophagus [20, 21]. The ARTDECO and CONCORDE trials have convincingly demonstrated a lack of benefit with dose escalation for unresectable esophageal cancer (SCC and adenocarcinoma) [22, 23]. Additionally, the Checkmate 577 trial indicated a disease-free survival benefit with the addition of postoperative pembrolizumab in patients with residual disease after CRT [24]. Over the past several decades, treatment advances have likewise caused a steady improvement in survival outcomes for esophageal cancer in Asian, European, and American populations [6–8, 25]. With improvements in long-term survival and cure rates, the effect of treatment-related morbidity and long-term quality of life have become increasingly relevant for esophageal cancer patients.

Proton therapy has repeatedly shown dosimetric advantages compared with photon-based radiation therapy (including IMRT and volumetric modulated arc therapy), with substantially reduced doses to the heart and lungs. Shiraishi et al [15] compared IMRT and proton therapy dosimetry in a large cohort of treated patients and showed that proton therapy reduced the mean heart dose by 40%. Compared with IMRT, proton therapy reduced the mean heart V5, V10, V20, V30, and V40 by 55%, 54%, 45%, 33%, and 18%, respectively [15]. Wang et al [26] compared proton therapy and IMRT treatment plans for 55 esophageal cancer patients and showed a reduction in mean lung dose, lung V5, V10, and V20 of 32%, 43%, 31%, and 25%, respectively, using passive-scatter proton beam therapy.

### Disease Control Outcomes

Limited data exist on disease control and survival outcomes among patients treated with proton CRT for esophageal cancer (see **Table 5** [14, 27–30]). Altogether, only a few hundred patients with disease control outcomes for proton CRT have been reported in the literature, most of whom come from a single institution [31, 32]. As we explore the benefits of proton therapy for toxicity reduction, it is important to ensure that disease control outcomes remain at least equivalent to those observed with photon radiation therapy. While reliable comparisons across datasets are a challenge, particularly when including patients treated with bimodality and trimodality therapy as well as SCC and adenocarcinoma histologies, our series appears favorable compared with contemporary esophageal cancer outcomes, with 3-year progression-free and overall survival rates of 66% and 55%, respectively. In the CROSS trial, investigators reported 3-year progression-free and overall survival rates of 51% and 58% in their trimodality cohort [20]. NEOCRTEC5010 investigators reported 3-year overall and disease-free survival approaching 70% for the trimodalilty cohort (SCC only) [21]. By comparison, the ARTDECO trial investigators reported a 3-year overall survival rate of 42% in the bimodality, standard-dose arm (including adenocarcinoma and SCC) [22]. Lin et al [14] showed equivalent disease control and survival outcomes among patients treated with protons and photons in their phase II randomized trial (including bimodality and trimodality treatment).

**Table 5.** Literature review of clinical outcomes in patients with esophageal cancer treated with proton radiation therapy and concurrent chemotherapy.

**Series**	**Institution**	**Dates**	**Study type**	**PBT Patients, n**	**PBT technique**	**Histology**	**OS, y (%)**
Xi et al. [27]	MDACC	2007–2014	Retrospective	132	PS, PBS	ACA, SCC	5 (42)
Ishikawa et al. [28]	Tsukuba	2008–2012	Retrospective	40	PS	SCC	3 (70)
Prayongrat et al. [29]	MDACC	2011–2016	Retrospective	19	PBS	ACA, SCC	2 (88)
Zeng et al. [30]	UW	2014–2015	Retrospective	13	PBS	ACA, SCC	N/A
Lin et al. [14]	MDACC	2019–2021	Phase II Randomized	46	PS, PBS	ACA, SCC	3 (45)
Rutenberg et al. (current)	UF	2010–2021	Retrospective	17	PS, PBS	ACA, SCC	3 (55)

Abbreviations: PBT, proton beam therapy; OS, overall survival; MDACC, MD Anderson Cancer Center; PS, passive scattered; PBS, pencil-beam scanning; ACA, adenocarcinoma; SCC, squamous cell carcinoma; UW, University of Washington; N/A, not available; UF, University of Florida.

A phase II randomized trial comparing proton therapy with IMRT for the treatment of esophageal cancer with or without surgery demonstrated that the dosimetric benefits of protons translate into improved clinical outcomes with reduced cardiopulmonary toxicity [14]. One hundred seven patients were randomized to IMRT or proton therapy (80% were treated with passive scattering) with concurrent chemotherapy. Progression-free survival and a novel cumulative cardiopulmonary toxicity score weighted for severity (total toxicity burden) served as coprimary endpoints. As noted above, the investigators reported no difference in disease control or survival endpoints; however, they showed significantly increased CRT-related cardiopulmonary toxicities (2.3-fold) and postoperative cardiopulmonary complications (7.6-fold) with IMRT compared with protons. These results led to an ongoing cooperative group phase III randomized trial, NRG GI006, comparing proton and photon radiation therapy using the same endpoints.

### Patterns of Failure

Owing to the increased sensitivity to organ motion and variations in tissue density associated with the treatment of esophageal cancer, careful attention to beam angles and treatment planning parameters are required to ensure robust proton planning and accurate proton delivery. Numerous strategies have been evaluated and are recommended to achieve effective delivery [32]. Clinical outcomes data are necessary to confirm the expected results. To assess the proton delivery accuracy, we analyzed local control and treatment response to CRT. In our series, 3-year local control was 85%. Two patients (12%) had local failures after refusing surgical resection after CRT. They both had a complete clinical response to radiation therapy but recurred at the site of the initial primary disease. The first proton series from the MD Anderson Cancer Center reported outcomes among 62 esophageal cancer patients treated with double-scattered proton therapy and concurrent chemotherapy [16]. The 26 patients in their trimodality cohort reported no in-field failures (primary site or lymph node). Among the 33 patients who received chemotherapy and proton beam therapy without surgery, 10 failures or persistent disease were at the primary site (30%), and 3 were in-field nodal failures [16].

Similarly, in our series, no patient treated with trimodality therapy had an in-field failure (0/9), while 2 patients (25%) treated with definitive CRT had local disease recurrence at the primary site. Of these, 1 patient underwent salvage esophagectomy, and the other refused salvage surgery and developed progressive disease. Three patients in our cohort (2 trimodality and 1 bimodality) developed distant metastatic disease at the time of death or last follow-up.

Of note, all but 1 patient in our series and all the patients in the MD Anderson Cancer Center series detailing failure patterns were treated with passively scattered proton beam therapy. The 1 patient in our series treated with PBS had a very large target volume requiring matching fields and was therefore selected for PBS. Although PBS delivery improves target dose conformality and proximal beam modulation and enables integrated boosts, it has the added complexity and uncertainty of the interplay effect between target or tissue motion and scanning beam delivery [33]. While most clinical outcomes reported for esophageal cancer treatment with proton therapy have used passive scattering, radiation oncologists at proton facilities increasingly use only PBS [29, 30]. Currently, no dosimetric studies have been published comparing passively scattered and PBS protons for esophageal cancer. At the University of Florida, we continue to use passively scattered protons, when possible, for esophageal cancer and reserve PBS for targets overly large or too complex for passive scattering.

### Toxicity

Esophagitis is the most common adverse event associated with proton therapy of the esophagus, which is unsurprising considering the affected organ is also the radiation target. In our series, 59% of patients experienced grade 2 (29%) or grade 3 (29%) esophagitis. Beyond the expected incidence of esophagitis, the only severe acute, nonhematologic treatment-related toxicities included 2 patients (12%) with grade 3 dehydration and 1 patient each with grade 3 nausea and vomiting (6% each).

Along with the obvious benefits of proton therapy in reducing heart and lung toxicity, additional benefits may exist in the form of reduced acute hematologic toxicity, particularly leukopenia and lymphopenia. Owing to the sensitivity of white blood cells to ionizing radiation, reduced exposure of the heart, lung, and bone marrow to low and moderate radiation doses with protons can reduce the risk of severe leukopenia [17, 34, 35]. In a propensity-matched analysis, Shiraishi et al showed a significant decrease in the incidence of grade 4 lymphopenia in esophageal cancer patients treated with protons compared with IMRT. Beginning at week 3 of CRT, they reported a doubling of the rate of grade 4 lymphopenia in the IMRT group compared with the proton group, which by week 5 of CRT reached rates of 40.4% and 17.6%, respectively. Several studies have demonstrated the dosimetric correlation between the thoracic vertebra and cardiopulmonary volume on the severity of leukopenia and/or lymphopenia, consistently showing the effect of increasing volumes of low and intermediate radiation dose (5-30 Gy) on these tissues [36–38]. Of note, the study by Shiraishi et al and others on esophageal cancer and other solid tumors have shown a correlation between severe leukopenia and lymphopenia during CRT and decreased disease control and overall survival [27, 39–41].

Two patients in our cohort experienced grade 3 or higher late radiation-associated toxicities. A grade 5 tracheoesophageal fistula with hemorrhage developed in a patient with an esophageal stent placed 3 months after completing CRT to 64.8 GyRBE for inoperable disease. It is likely that a combination of high-dose CRT and the esophageal stent contributed to the fistula and hemorrhage. The other severe late toxicity was grade 3 esophageal stenosis requiring a feeding tube. This occurred in a patient who received 54 GyRBE and refused surgical resection.

The current study has important limitations, including those inherent to all retrospective reviews. This includes patient selection bias and likely underreporting of treatment-associated toxicities. One potential source of bias in this population includes a cohort in which many patients sought proton radiation therapy as a destination medical treatment. This is typically a population with better resources and social support, which may affect oncology outcomes and limit the generalizability of outcomes. The small sample size limits our ability to draw firm conclusions or perform extensive statistical analyses. In addition, our hematologic toxicity analysis was limited because many patients had complete blood counts without a differential white blood cell count. Another limitation of our study is the lack of prospectively obtained quality-of-life metrics.

There are increasing data supporting the use of proton therapy for esophageal cancer, indicating very good safety and efficacy. High-level retrospective data already suggest a benefit of proton radiation therapy compared with photon-based radiation therapy for cardiopulmonary toxicity. We will await ongoing phase III studies to confirm these results.
