# Supplementary material for: Proton Therapy With Concurrent Chemotherapy for Thoracic Esophageal Cancer: Toxicity, Disease Control, and Survival Outcomes
Source: Int J Part Ther. 2022 Dec 19;9(3):18–29. doi: 10.14338/IJPT-22-00021.1 (PMC9875824; doi:10.14338/IJPT-22-00021.1)
Supplement: Supplementary file 1 [file ijpt-09-03-05_s01.docx]

| **Supplemental Table.** Patient Comorbidities Prior to Esophageal Cancer Diagnosis (n= 17) | |
| --- | --- |
| Comorbid Condition | No. of Patients |
| Melanoma | 1 |
| Coronary artery disease | 1 |
| High blook pressure | 9 |
| Hypothyroidism | 1 |
| Gastroesophageal reflux disease | 9 |
| Hiatal hernia | 3 |
| Hyperlipidemia | 5 |
| Prostate cancer | 2 |
| Thyroid disease | 2 |
| Diabetes mellitus | 2 |
| History of cerebrovascular accident | 1 |
| History of arteriovenous malformations | 1 |
| Arthritis | 2 |
| Renal Insufficiency/chronic kidney disease | 1 |
| Papillary renal cell carcinoma | 1 |
| Deep vein thrombosis | 1 |
| Heart failure | 1 |
| Asthma | 1 |
| Lynch syndrome | 1 |
| Chronic obstructive pulmonary disease | 1 |
| Depression/anxiety | 2 |
| Sjogren’s syndrome | 1 |
| Achalasia | 1 |
| Non-melanoma skin cancer | 1 |
| Non-Hodgkin lymphoma | 1 |
| Peptic ulcer disease | 1 |
