# Supplementary figures and images for: Proton Therapy With Concurrent Chemotherapy for Thoracic Esophageal Cancer: Toxicity, Disease Control, and Survival Outcomes
Source: Int J Part Ther. 2022 Dec 19;9(3):18–29. doi: 10.14338/IJPT-22-00021.1 (PMC9875824; doi:10.14338/IJPT-22-00021.1)

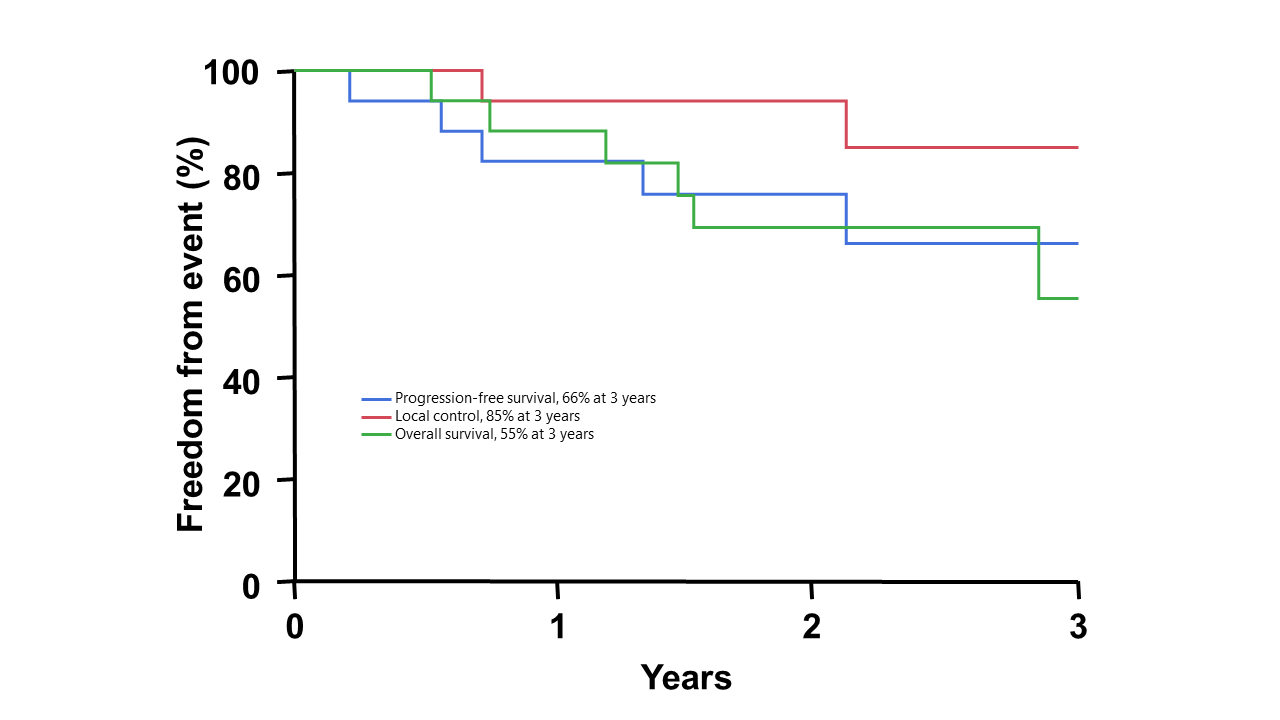

Supplement: Supplementary file 2 [file ijpt-09-03-05_s02.tif]
